# Effect of high-frequency repetitive transcranial magnetic stimulation on cognitive impairment in WD patients based on inverse probability weighting of propensity scores

**DOI:** 10.3389/fnins.2024.1375234

**Published:** 2024-04-10

**Authors:** Hong Chen, Xie Wang, Juan Zhang, Daojun Xie

**Affiliations:** ^1^The First Clinical Mdical College of Anhui University of Chinese Medicine, Hefei, China; ^2^Department of Neurology, The First Affiliated Hospital of Anhui University of Traditional Chinese Medicine, Hefei, China

**Keywords:** high frequency repetitive transcranial magnetic stimulation, propensity score, inverse probability weighting, Wilson disease, cognitive impairment

## Abstract

**Background:**

Hepatolenticular degeneration [Wilson disease (WD)] is an autosomal recessive metabolic disease characterized by copper metabolism disorder. Cognitive impairment is a key neuropsychiatric symptom of WD. At present, there is no effective treatment for WD-related cognitive impairment.

**Methods:**

In this study, high-frequency repetitive transcranial magnetic stimulation (rTMS) was used to treat WD-related cognitive impairment, and inverse probability weighting of propensity scores was used to correct for confounding factors. The Mini-Mental State Examination (MMSE), Montreal Cognitive Assessment (MoCA), Auditory Verbal Learning Test (AVLT), Boston Naming Test (BNT), Clock Drawing Test (CDT) and Trail Making Test (TMT) were used to evaluate overall cognition and specific cognitive domains.

**Results:**

The MMSE, MoCA and CDT scores after treatment were significantly different from those before treatment (MMSE: before adjustment: OR = 1.404, 95% CI: 1.271–1.537; after adjustment: OR = 1.381, 95% CI: 1.265–1.497, *p* < 0.001; MoCA: before adjustment: OR = 1.306, 95% CI: 1.122–1.490; after adjustment: OR = 1.286, 95% CI: 1.104; AVLT: OR = 1.161, 95% CI: 1.074–1.248; after adjustment: OR = 1.145, 95% CI: 1.068–1.222, *p* < 0.05; CDT: OR = 1.524, 95% CI: 1.303–1.745; after adjustment: OR = 1.518, 95% CI: 1.294–1.742, *p* < 0.001). The BNT and TMT scores after adjustment were not significantly different from those before adjustment (BNT: before adjustment: OR = 1.048, 95% CI: 0.877–1.219; after adjustment: OR = 1.026, 95% CI: 0.863–1.189, *p* > 0.05; TMT: before adjustment: OR = 0.816, 95% CI: 1.122–1.490; after adjustment: OR = 0.791, 95% CI: 0.406–1.176, *p* > 0.05).

**Conclusion:**

High-frequency rTMS can effectively improve cognitive impairment, especially memory and visuospatial ability, in WD patients. The incidence of side effects is low, and the safety is good.

## Introduction

Wilson disease (WD) is an autosomal recessive metabolic disease characterized by copper metabolism disorder caused by ATP7B gene mutation. Substantial copper deposits in the liver, brain and other organs cause liver function damage and neurological symptoms (Członkowska et al., 2018). The mental symptoms of WD mainly include emotion, behavior, personality, anxiety and cognitive impairment, among which cognitive impairment is a key neuropsychiatric symptom (Frota et al., 2009). A survey showed that nearly 60% of WD patients had varying degrees of cognitive impairment (Wang et al., 2019). The onset of WD is slow and insidious. With the continuous deposition of copper, the disease gradually progresses.

Transcranial magnetic stimulation (TMS) is a noninvasive neuromodulation technique that transmits magnetic pulses at a specific frequency to specific tissues and regions of the brain through coils placed on the scalp (Latorre et al., 2019). Studies have shown that intermittent pulse stimulation can excite the cerebral cortex to a certain extent, while continuous pulse stimulation can inhibit the cortex (Suppa et al., 2011; Bologna et al., 2020). In addition, theta burst TMS, a new treatment method characterized by several burst pulse stimuli, has the advantages of short stimulation time and low stimulation intensity and has achieved efficacy in a variety of nervous system diseases, such as motor, language, and cognitive impairment disorders. However, due to its relatively short time in clinical use, its efficacy and safety still need further study (Ko et al., 2008). Repetitive transcranial magnetic stimulation (rTMS) has been shown to significantly improve cognition, apathy, memory and language, especially in the early stage of the disease (Di Lazzaro et al., 2021). To date, it has been widely used to treat cognitive impairment caused by a variety of conditions, including Alzheimer’s disease (AD), Parkinson’s disease (PD), and poststroke cognitive impairment (Koch et al., 2018; Trung et al., 2019; Li et al., 2023).

Treatments of the neuropsychiatric symptoms of WD and the efficacy of those treatments have been reported. The main treatment for tremor symptoms is β receptor blockers, among which phenobarbital is most common; levodopa and anticholinergic drugs are the main drugs used to treat dystonia; zolpidem and botulinum toxin are the main drugs used to treat dysarthria, and tetraphenylpromazine is the main treatment for chorea and hand-foot attention-deficit/hyperactivity disorder (ADHD; Litwin et al., 2023). To date, no drugs have been recommended for the treatment of WD-related cognitive impairment. Many studies have shown that anti-copper therapy can reduce copper deposition in the liver and brain, increase urinary copper content, and improve cognitive impairment in WD patients (Wang et al., 2023; Zhang et al., 2024). An effective and safe means of nerve regulation, TMS has achieved curative effects in the treatment of WD complicated with dystonia (Hao et al., 2021) and plays an important role in improving cognitive impairment with a variety of causes. However, there are few relevant reports on the treatment of WD complicated with cognitive impairment. In his study, high-frequency rTMS was used as an intervention for WD-related cognitive impairment, and the inverse probability weighting method of propensity scores was used to correct the bias caused by differences in multiple covariates. The causal relationship between processing factors and the effect value was further investigated (Carry et al., 2021) to clarify the exact effect of TMS on WD-related cognitive impairment and to provide a basis for the treatment of WD-related cognitive impairment in the clinic.

## Materials and methods

### Participants

The researchers recruited 80 patients with WD-related cognitive impairment from the Department of Neurology, the First Affiliated Hospital of Anhui University of Traditional Chinese Medicine.

### Ethics and informed consent

The ethical principles of this study are consistent with the Helsinki Declaration, good practice guidelines, and local regulatory requirements. All participants signed an informed consent form, and the study was approved by the Ethics Committee of the First Affiliated Hospital of Anhui University of Traditional Chinese Medicine.

### Diagnostic criteria

The diagnostic criteria for WD in this study were taken from the diagnostic guidelines for WD (European Association for Study of Liver, 2012) formulated by the European Association for the Study of the Liver from 2012. The diagnostic criteria for cognitive impairment were taken from the Diagnostic and Statistical Manual for Mental Disorders, Fifth Edition (DSM-5; Messent, 2013), of the American Psychiatric Association from 2013 and the comprehensive assessment of Chinese guidelines for the diagnosis and management of cognitive impairment and dementia (Jia et al., 2011), which were developed under the guidance of the academic group of the neurology branch of the Chinese Medical Association in 2011. The criteria were as follows: ① Patient self-reports or informant reports or a diagnosis of cognitive impairment from a clinical experienced doctor; ② objective evidence of impairment in one or more cognitive domains of cognitive tests; and ③ with or without varying degrees of impairment in complex activities of daily living.

### Inclusion and exclusion criteria

The inclusion criteria were as follows: ① met the diagnostic criteria for WD and cognitive impairment; ② voluntarily participated in the trial and signed the informed consent form; ③ were 18–35 years old; ④ had a specific educational level; sufficient visual, auditory and linguistic discrimination; no obvious obstacles in reading and writing simple sentences; and the ability to complete the relevant scale assessments; and ⑤ had a Mini Mental State Examination (MMSE; Zhang et al., 1999; Honig et al., 2018; Arevalo-Rodriguez et al., 2021) score ≤ 26分 (a score of 20–26 indicated mild cognitive impairment) and a Montreal Cognitive Assessment Scale (MoCA; Lu et al., 2011) score ≤ 25分. The exclusion criteria were as follows: ① secondary epilepsy or epileptic waves on EEG; ② a history of mental illness or suicidal tendencies and an inability to complete the relevant clinical assessments without guardianship; ③ severe heart, liver or kidney dysfunction; ④ use of intelligence-improving drugs in the past 3 months or participation in other clinical trials within 3 months; ⑤ pregnancy or lactation; ⑥ intracranial metal internal fixation; and ⑦ other circumstances that were determined to be unsuitable for inclusion after judgment.

### Research method

All participants in this study were from the Department of Neurology, the First Affiliated Hospital of Anhui University of traditional Chinese medicine. All patients who met the study criteria were randomly assigned to the treatment group (TG) or the control group (CG) according to the ratio of 1:1. The TG group was treated with rTMS. The stimulation parameters were 10 Hz (high frequency), and the duration was 20 min. Stimulation site: bilateral left and right dorsolateral prefrontal cortex (DLPFC), 10 min each time, 5 times a week for 4 weeks. CG group was given the same pseudo stimulation as TG group. At the same time, attention was paid to avoid the coil induced brain power generation, which only made the patients feel subjective TMS. All patients were treated at the same time of the day. During the treatment, the subjects sat on the chair, relaxed their arms, and kept the surrounding environment quiet without noise interference. Through the above methods, the influence of confounding factors on the research results can be reduced.

### Assessment methods and indicators

The participants were evaluated at two time points: before enrolment (pre) and after the fourth week of treatment (post). Each participant’s assessments were conducted on the same day. The evaluation indicators included the following: (1) Mini Mental State Examination (MMSE): The overall cognitive function of patients with WD-related cognitive impairment was evaluated with the MMSE (Li et al., 2016), with a total possible score of 30 points. Higher scores indicate better overall cognitive function. (2) Montreal Cognitive Assessment (MoCA): The MoCA (Hsu et al., 2015), which includes 11 items, including the alternative connection test, visual space and executive function (cube, clock), naming, memory, attention, sentence repetition, word fluency, abstraction, delayed recall and orientation, was used to assess the overall cognitive level of patients. The evaluation was completed by the doctors in charge of the neurology department, and the total scores before and after treatment were compared. (3) Specific cognitive domain function assessment: Different scales were used to assess the cognitive domain impairment of subjects in four specific areas. The Auditory Verbal Learning test (AVLT; Guo et al., 2009) was used to assess memory, the Boston Naming Test (BNT; Madore et al., 2022) was used to assess language function, the Clock Drawing Test (CDT; Suzuki et al., 2019) was used to assess visuospatial ability, and the Trail Making Test (TMT, A + B; Guo, 2022) was used to assess executive function.

### Statistical analysis

Propensity score and inverse probability weighting methods were used to adjust for the imbalance of covariates in patients with WD-related cognitive impairment. We performed propensity score matching on general patient data and baseline clinical data to evaluate the probability of each patient receiving TMS therapy. Next, inverse probability weighting was applied to the obtained probability, and confounding factors were corrected for to evaluate the impact of TMS on multiple cognitive function scores in individuals with WD-related cognitive impairment.

## Results

### Baseline characteristics of population data

We recruited 80 patients with WD-related cognitive impairment who met the inclusion criteria. Among them, 2 patients had severe liver and renal dysfunction, 6 patients had recently taken cognitive-related drugs, and 4 patients refused to participate in the study. Two patients in the treatment group and 3 patients in the control group withdrew from the study. A total of 65 patients were ultimately included in the analysis. The baseline demographic data are shown in Table 1. Compared with those in the control group, the patients in the treatment group were relatively older, and the course of disease was relatively shorter, although the differences were not statistically significant (*p* > 0.05). The age and TMT score before treatment (SMD > 01) suggested that there was a variable imbalance.

**Table 1:** General information and baseline data before treatment.

	TG	CG	*P*	SMD
Subjects	34	34		
Dropped out	2	3		
Completed	32	31		
Age (year)	24.65 (4.28)	23.34 (4.66)	0.253	0.291
Gender (male)	17 (54.8%)	18 (56.2%)	1.000	0.028
Course (year)	6.00 (2.34)	6.63 (2.34)	0.291	0.268
MMSE pre	20.65 (2.56)	20.72 (2.11)	0.901	0.031
MoCA pre	20.84 (2.03)	20.84 (2.23)	0.993	0.002
AVLT-H pre	31.58 (3.23)	31.44 (2.68)	0.849	0.048
BNT pre	20.94 (2.54)	20.97 (2.02)	0.954	0.014
CDT pre	20.32 (1.97)	20.41 (2.50)	0.883	0.037
TMT pre	184.94 (7.25)	185.75 (6.55)	0.641	0.118

### Prediction probability of the propensity score model

**Figure 1:**
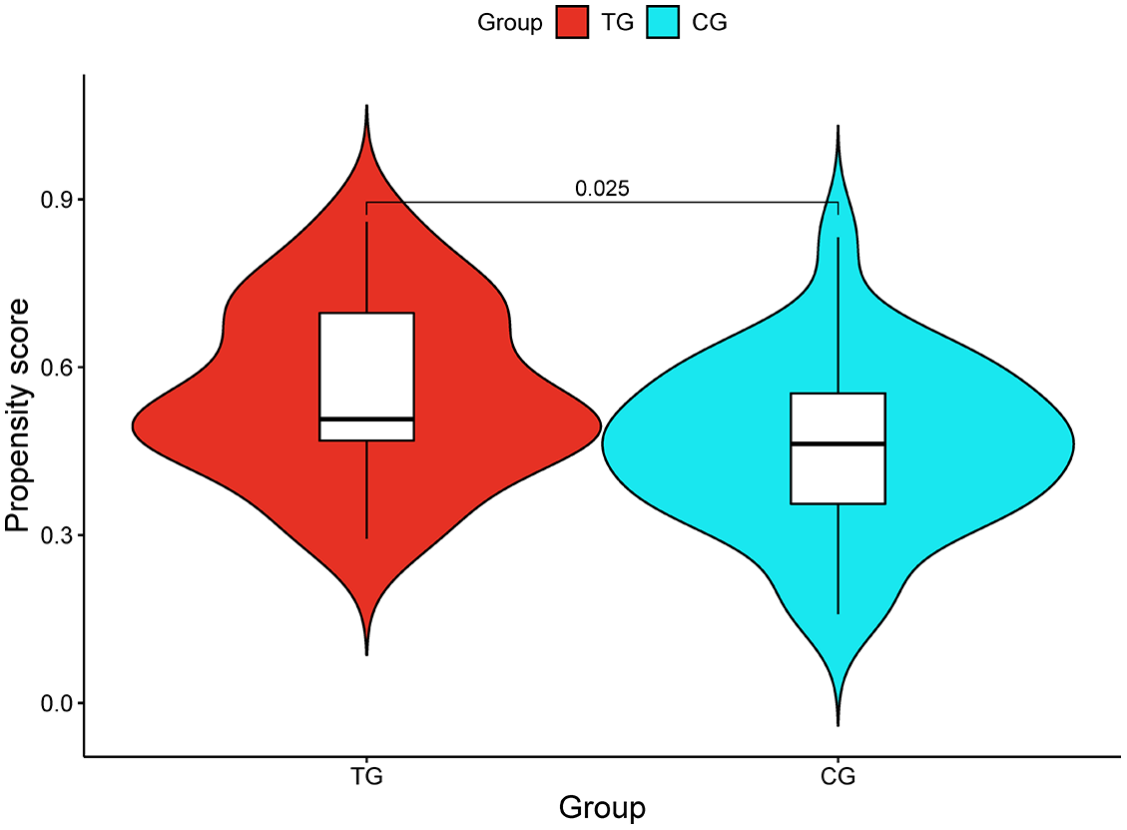
Violin diagram of propensity score model in predicting the probability of cognitive impairment patients with Wilson disease.

We used the propensity score model to predict the probability of all patients having WD complicated with cognitive impairment, and each patient was given a specific propensity score. The median propensity score of the TG was 0.507 (IQR: 0.460–0.701), and the median propensity score of the CG was 0.463 (IQR: 0.349–0.564). A violin plot was drawn to visualize the propensity score results of the two groups (Figure 1).

### Inverse probability weighting method to correct for confounding factors

**Figure 2:**
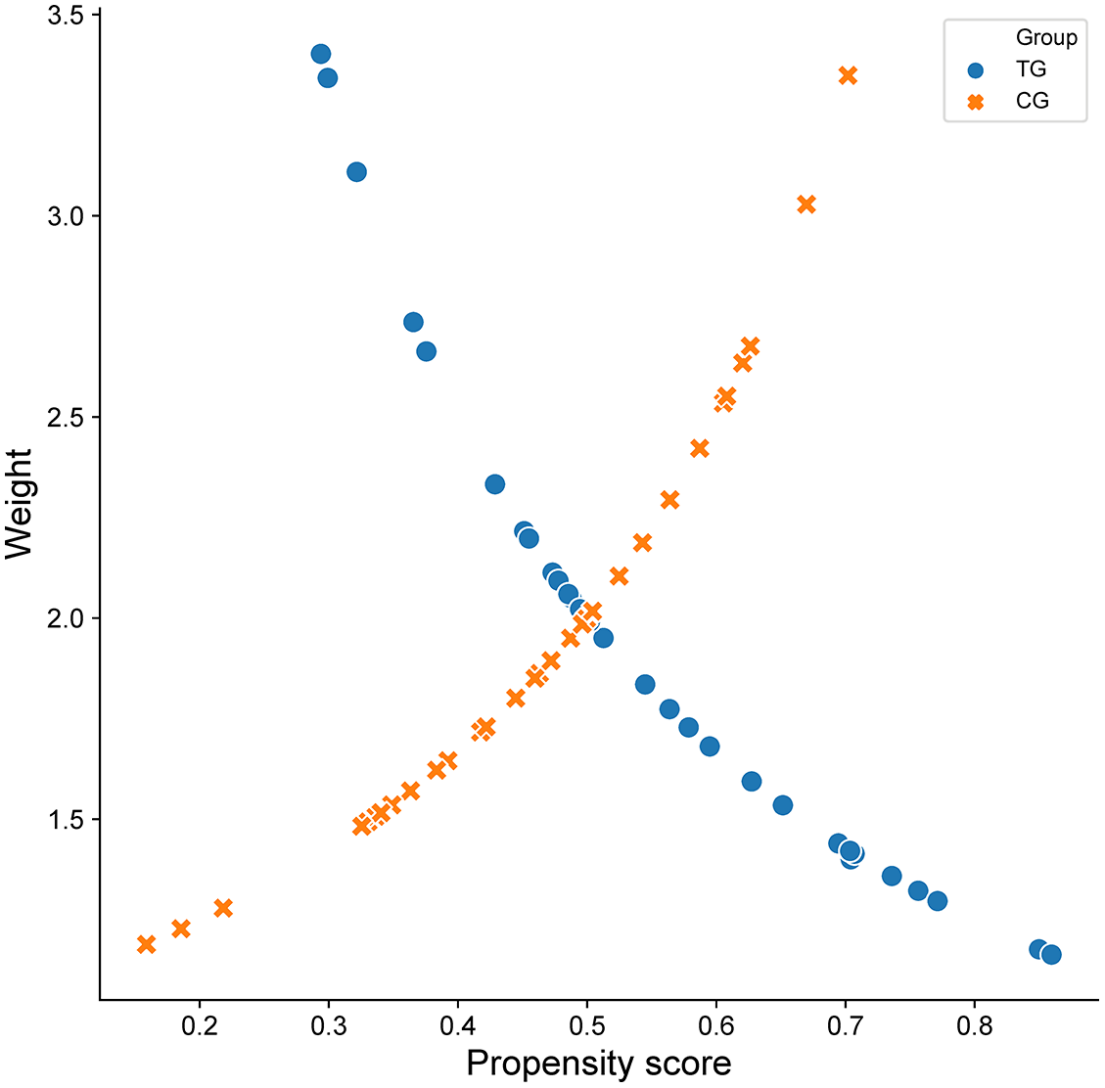
Scatter diagram of propensity score and inverse probability weighted data in two groups of patients with Wilson disease complicated with cognitive impairment.

**Figure 3:**
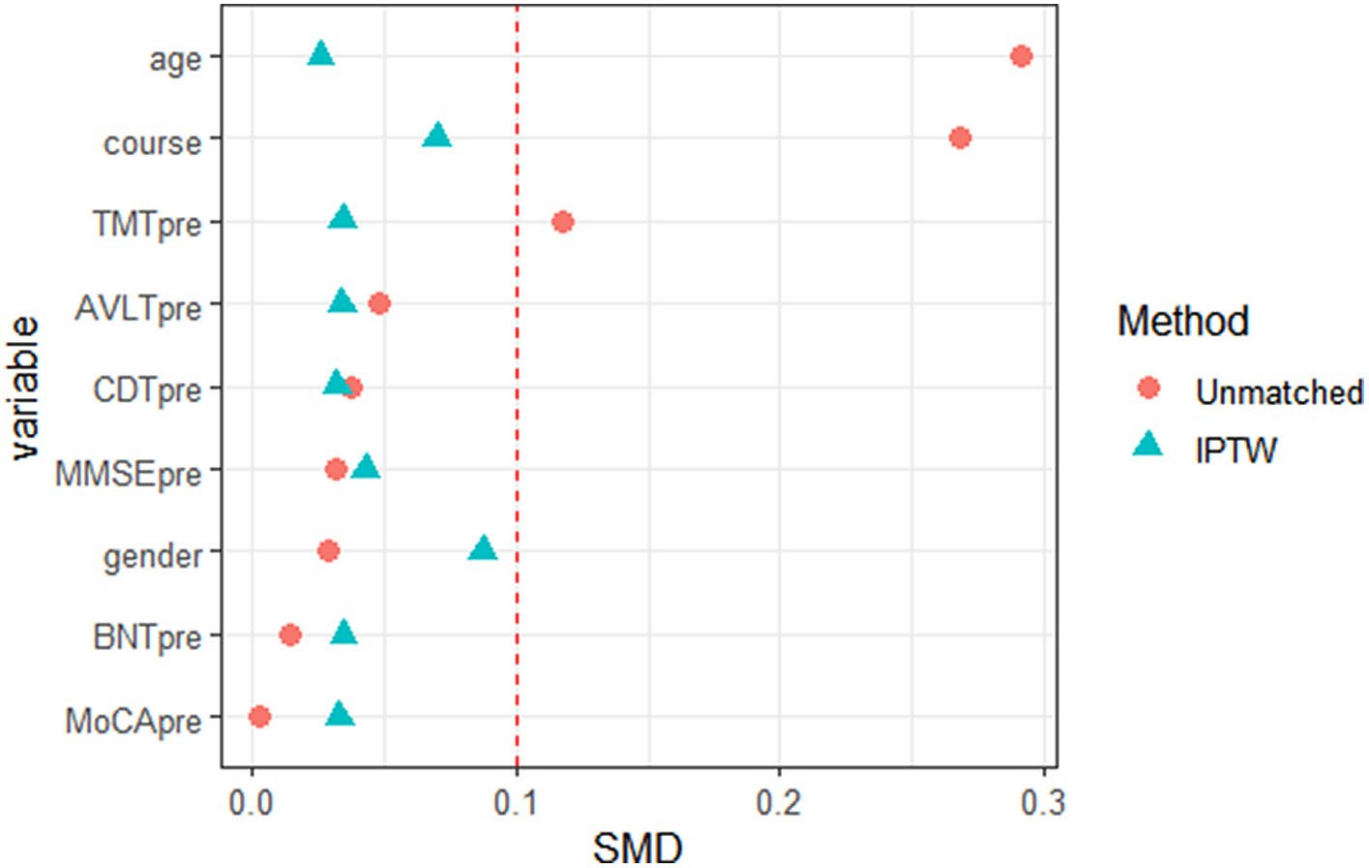
Data distribution diagram of baseline and pre-treatment data of Wilson disease patients with cognitive impairment before and after inverse probability weighting (red icon indicates data distribution before inverse probability weighting, and green icon indicates data distribution after inverse probability weighting).

According to the propensity score of WD patients with cognitive impairment, the data were further weighted by inverse probability, and each patient in the TG and CG was given a corresponding weight. The weight composition and trends of the two groups were similar. When both groups had a high prediction probability, the weight of the TG was lower than that of the CG. In contrast, when the weights of the two groups of patients were the same, the CG had a greater prediction probability than the TG (Figure 2). The weighted data were analyzed, and the results are shown in Table 2. After correction, all *p* values were >0.05, and the SMD was <10%, indicating that the imbalance caused by confounding factors in the baseline and preoperative data had been corrected (Figure 3).

**Table 2:** Statistics of baseline and clinical data of Wilson disease patients with cognitive impairment after inverse probability weighting.

	TG	CG	*P*	SMD
Age (year)	24.10 (4.90)	23.98 (4.35)	0.925	0.026
Gender (male)	17.4 (54.3%)	17.8 (57.4%)	0.747	0.087
Course (year)	6.44 (2.31)	6.29 (2.21)	0.781	0.070
MMSE post	20.63 (2.13)	20.73 (2.50)	0.867	0.043
MoCA post	20.76 (2.21)	20.83 (2.11)	0.904	0.033
AVLT-H post	31.47 (2.66)	31.37 (3.14)	0.897	0.034
BNT post	20.93 (2.11)	20.85 (2.47)	0.897	0.034
CDT post	20.31 (2.47)	20.24 (1.95)	0.902	0.032
TMT post	185.62 (6.34)	185.85 (7.43)	0.898	0.034

### Evaluation of cognitive function by the inverse probability weighting method

**Figure 4:**
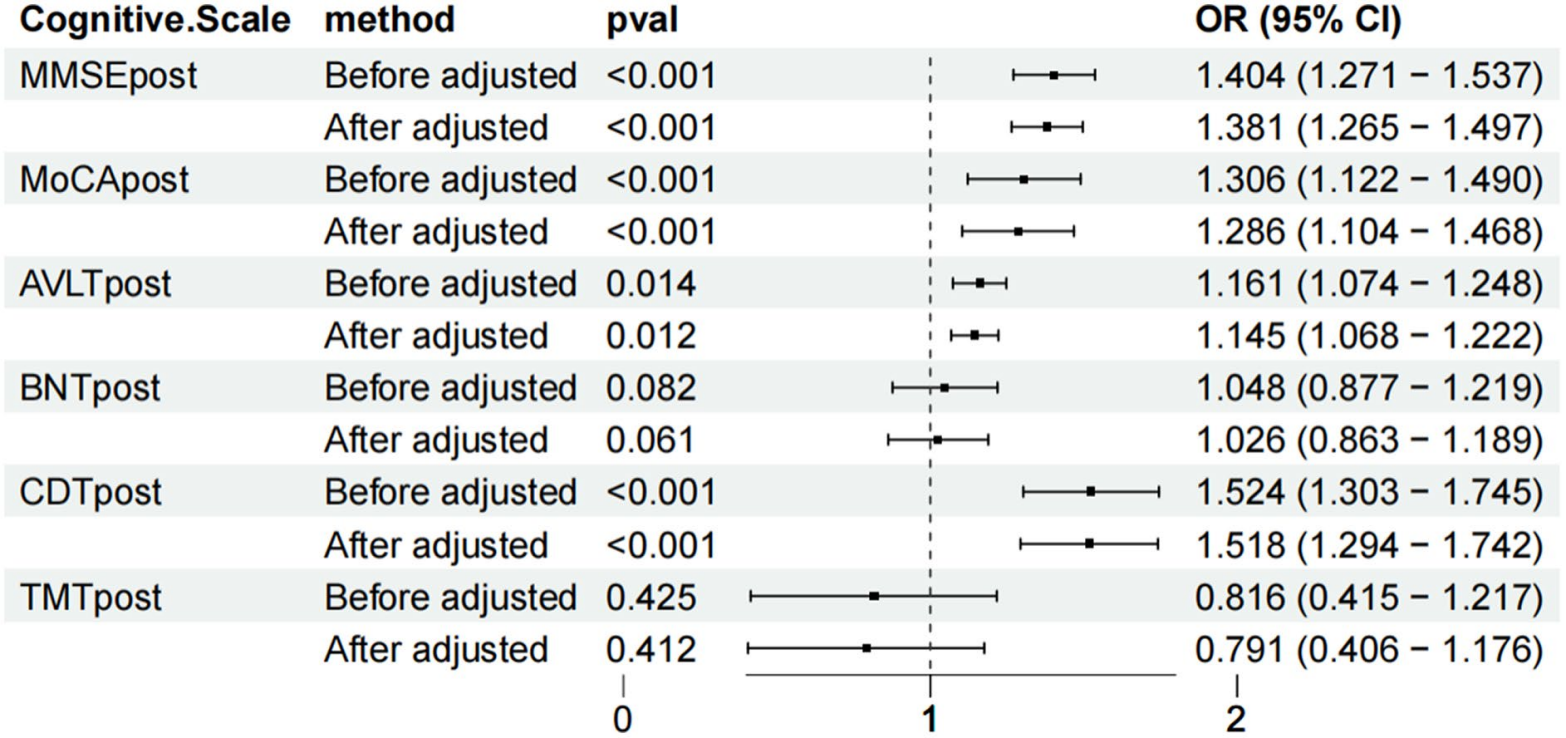
Forest chart of the scale score weighted statistics of the two groups of patients with Wilson disease complicated with cognitive impairment after treatment.

The MMSE, MoCA, AVLT, BNT, CDT and TMT scores in the TG and CG of WD patients with cognitive impairment before and after weighting were compared, and the MMSE, MoCA and CDT scores were significantly different before and after adjustment (MMSE: before adjustment: OR = 1.404, 95% CI: 1.271–1.537, after adjustment: OR = 1.381, 95% CI: 1.265–1.497, *p* < 0.001; MoCA: before adjustment: OR = 1.306, 95% CI: 1.122–1.490, after adjustment: OR = 1.286, 95% CI: 1.104–1.468, *p* < 0.001; AVLT: OR = 1.161, 95% CI: CDT: OR = 1.524, 95% CI: 1.303–1.745, after adjustment: OR = 1.518, 95% CI: 1.294–1.742, *p* < 0.001), suggesting that the results are relatively robust. After treatment, the BNT and TMT scores were not significantly different before and after adjustment (BNT: before adjustment: OR = 1.048, 95% CI: 0.877–1.219; after adjustment: OR = 1.026, 95% CI: 0.863–1.189, *p* > 0.05; TMT: before adjustment: OR = 0.816, 95% CI: 1.122–1.490; after adjustment: OR = 0.791, 95% CI: 0.406–1.176, *p* > 0.05; Figure 4).

### Side effects

**Figure 5:**
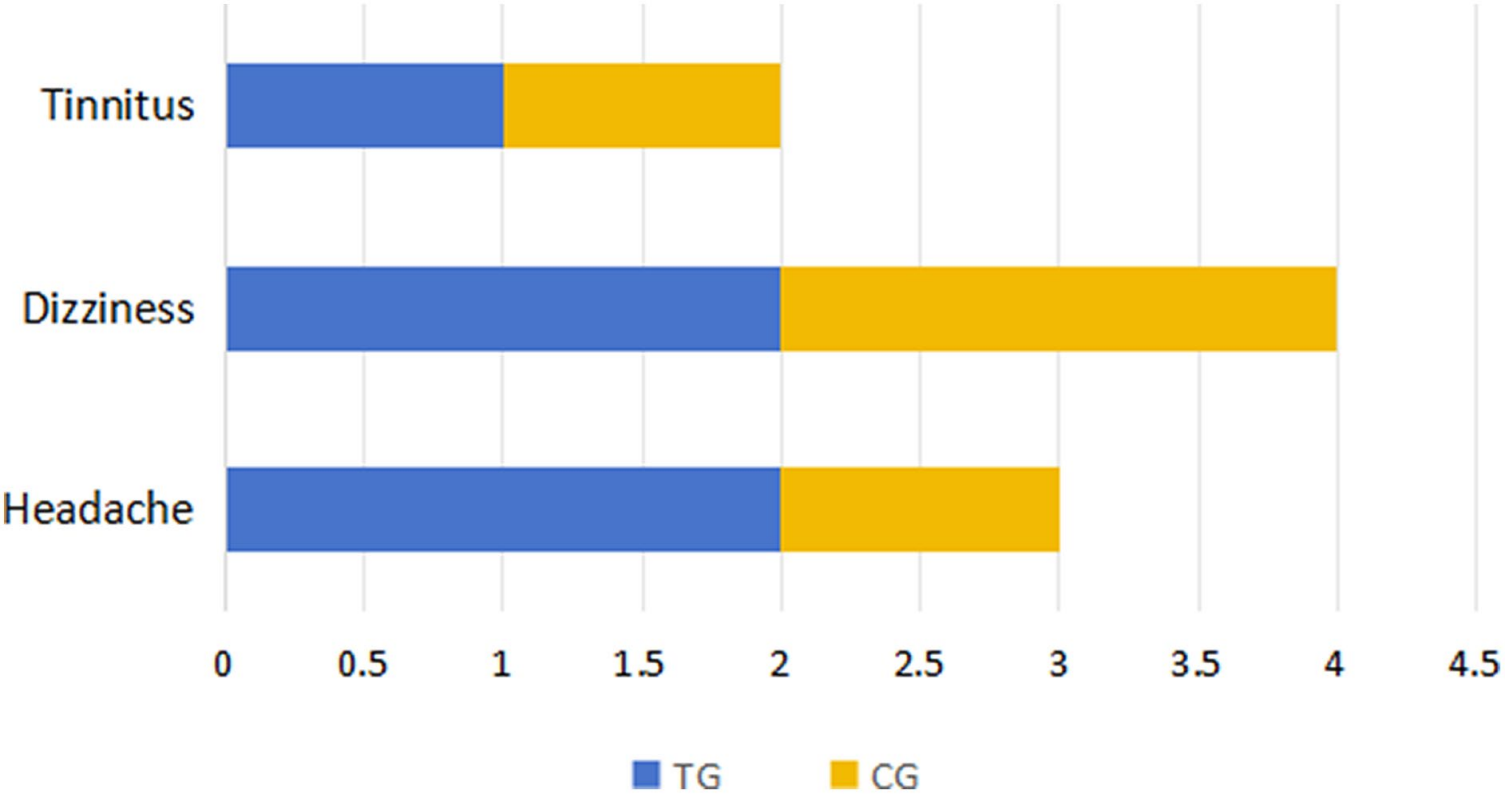
Statistical histogram of side effects in two groups.

The adverse reactions of the patients in both groups were counted to evaluate the safety of the treatment. The main adverse reactions were dizziness, headache and tinnitus, with 2 cases of dizziness, 2 cases of headache and 1 case of tinnitus observed in the TG; the CG reported 1 case of dizziness, 2 cases of headache and 1 case of tinnitus (Figure 5).

## Discussion

This study used propensity score and inverse probability weighting methods to investigate the efficacy of high-frequency rTMS in treating cognitive impairment in WD patients for the first time while controlling for multiple confounding factors, such as sex, age and disease course. Studies have shown that high-frequency rTMS can effectively improve cognitive impairment, especially memory and visuospatial ability, in WD patients. In addition, the language function and executive ability of the patients did not significantly improve. The incidence of side effects was low, and the safety was relatively high.

The treatment of cognitive impairment in WD patients mainly includes copper chelators (including penicillamine, sodium dimercaptopropane sulfonate, and trientine) to increase urinary copper excretion and zinc agents to reduce copper absorption (Schilsky, 2017). The use of bis-choline tetrathiomolybdate, trientine and methanobactin (TTM) as a potential WD therapeutic treatment (Litwin et al., 2019) is still in the experimental stage. Gene therapy targeting ATP7B may become an effective treatment for WD in the future (Li et al., 2013). Long-term drug treatment may aggravate patients’ mental and neurological symptoms and even cause liver and kidney dysfunction, bone marrow suppression, skin rash and other side effects (Poujois and Woimant, 2018). According to the latest guidelines, no drugs have been approved for the treatment of WD-related cognitive impairment (Lucena-Valera et al., 2023). Therefore, it is becoming increasingly urgent to explore safe and effective treatments for WD-related cognitive impairment.

TMS transmits a current to the cerebral cortex through coils with specific magnetic fields and pulses, causing depolarization of brain cell membranes and cross-synaptic depolarization or hyperpolarization of cortical neuron groups (Barker et al., 1985, 1987). The pulse with the strongest repetition frequency was used to separate short pauses without stimulation to achieve the best stimulation effect (Hamada et al., 2008). One study revealed that weekly rTMS therapy can effectively improve the working memory ability of patients with cognitive impairment and has a stable effect on the decline in cognitive status in patients with AD (Moussavi, 2022). In addition, TMS can effectively improve the overall cognitive ability of Parkinson’s disease patients with mild cognitive impairment (Trung et al., 2019). The observation of patients with cognitive impairment after stroke showed that TMS combined with cognitive training had a positive effect on the overall cognition, executive function and working memory of patients (Gao et al., 2023). A meta-analysis suggested that high-frequency TMS in the DLPFC can improve the overall cognitive function of patients with age-related neurodegenerative diseases (Miller et al., 2023). For patients with ADHD, rTMS can effectively improve sustained attention, processing speed and overall cognitive function (Chen et al., 2023). Many scholars have explored the potential mechanism by which TMS affects cognitive impairment. TMS reduced the downregulation of autophagy signals and enhanced the expression of synaptic plasticity-related factors, thereby alleviating the damage to hippocampal spatial cognition and synaptic plasticity in mice (Liu et al., 2023). Chronic high-frequency TMS may improve age-related cognitive impairment in mice by enhancing the neuronal excitability of hippocampal dentate gyrus granulosa cells (Zhu et al., 2020).

In this study, high-frequency rTMS improved the overall cognitive function, especially memory and visuospatial function, of patients with WD. The language function and executive ability of patients did not improve significantly. This may be related to the main pathological mechanism of WD. Due to the copper metabolism disorder caused by ATP7B mutation, most of the copper is deposited in the basal ganglia and cortex of the brain. Not only is the basal ganglia connected to the cortex, but the hippocampus is also widely connected to the cortex and basal ganglia. Therefore, changes in the circuits of the cortex and basal ganglia are directly related to cognitive function, thus explaining the motor and cognitive impairment of patients (Tian et al., 2023). TMS can affect WD-related cognitive impairment by stimulating closed neural circuits in the basal ganglia and cortex (DeLong and Wichmann, 2015; Aggarwal and Bhatt, 2020), which may be one of the potential mechanisms by which TMS improves the cognitive function of patients. However, there are no relevant studies on the specific mechanism of TMS in the treatment of WD-related cognitive impairment, and additional experimental and clinical studies are needed for further exploration and verification.

This study has several limitations. First, we only studied the effect of conventional high-frequency rTMS on patient cognition and did not compare different frequencies to obtain the best stimulation frequency. Second, due to the large number of cognitive-related scales, we only selected the scales that reflect the overall cognitive level and individual specific cognitive domains, which also leads to some deviation in the results. Third, we did not select relevant biomarkers to use to effectively evaluate the research indicators and efficacy. Fourth, due to the limitations of the study design, we were unable to observe these effects for a longer period to ensure the reliability of the curative effect. Therefore, in the future, it is necessary to establish a more rigorous research program, select a more comprehensive scale and related biomarkers, and perform a more detailed distinction and evaluation of each cognitive domain after a longer intervention cycle.

### Summary

To our knowledge, this is the first study on the effect of TMS on the cognitive ability of patients with WD-related cognitive impairment. Our research showed that high-frequency rTMS can effectively improve the overall cognitive level of patients, especially their memory and visuospatial ability, with good safety. This research scheme can be used as a supplementary treatment method to provide a reference for the treatment of WD-related cognitive impairment in the clinic.

## Data availability statement

The original contributions presented in the study are included in the article/supplementary material, further inquiries can be directed to the corresponding author.

## Ethics statement

The studies involving humans were approved by the Ethics Committee of the First Affiliated Hospital of Anhui University of traditional Chinese medicine. The studies were conducted in accordance with the local legislation and institutional requirements. Written informed consent for participation in this study was provided by the participants’ legal guardians/next of kin.

## Author contributions

HC: Conceptualization, Data curation, Methodology, Writing – original draft, Writing – review & editing. XW: Conceptualization, Formal analysis, Methodology, Project administration, Writing – original draft. JZ: Formal analysis, Funding acquisition, Methodology, Resources, Writing – review & editing. DX: Conceptualization, Formal Analysis, Funding acquisition, Project administration, Resources, Supervision, Writing – review & editing.
